# The Use of Monoclonal Antibody-Based Proprotein Convertase Subtilisin-Kexin Type 9 (PCSK9) Inhibitors in the Treatment of Hypercholesterolemia

**DOI:** 10.7759/cureus.25641

**Published:** 2022-06-03

**Authors:** Riya R Parikh, Frank Breve, Peter Magnusson, Payam Behzadi, Joseph Pergolizzi

**Affiliations:** 1 School of Pharmacy, Temple University, Philadelphia, USA; 2 School of Medical Sciences, Örebro University, Örebro, SWE; 3 Cardiology Research Unit, Department of Medicine, Karolinska Institutet, Stockholm, SWE; 4 Microbiology, Islamic Azad University, Shahr-e-Qods Branch, Tehran, IRN; 5 Clinical Research, Native Cardio Inc., Naples, USA

**Keywords:** ldl-c, low density lipoprotein cholesterol, cardiovascular, ascvd, cholesterol, ldl, alirocumab, evolocumab, monoclonal antibody, pcsk9 inhibitors

## Abstract

In this review, we evaluated several studies in the literature to analyze the benefits and deleterious effects of the use of monoclonal antibodies (MABs)-based proprotein convertase subtilisin-kexin type 9 (PCSK9) inhibitors in patients with hypercholesterolemia. Increased low-density lipoprotein cholesterol (LDL-C) levels lead to an increase in the risk of cardiovascular (CV) disease. Statins are the cornerstones of hypercholesterolemia treatment, but the patient response may often vary, and additional therapies may be needed to control the increased LDL-C levels. MABs bind to PCSK9 receptors, causing a reduction in LDL-C levels. MAB-based PCSK9 inhibitors such as alirocumab and evolocumab have been approved for use in hypercholesterolemia in combination with statins. Studies have suggested that both alirocumab and evolocumab are effective in lowering LDL-C levels, have favorable side effect profiles, and can be administered at convenient dosing intervals; however, further double-blind, randomized trials evaluating the long-term safety and efficacy of both the agents could assist with clinical decision-making.

## Introduction and background

It has been well established that atherosclerotic cardiovascular diseases (ASCVD) are one of the major causes of mortality around the world [1,2], mainly resulting from increased levels of cholesterol, specifically the low-density lipoprotein (LDL). It is one of the seven factors used by the 2021 American Heart Association (AHA) update to assess cardiovascular (CV) risks [3]. In this update, between 2013 and 2016, across both sexes, 69.6 million Americans were found to have low-density lipoprotein cholesterol (LDL-C) levels ≥130 mg/dL. A total cholesterol (TC) level of 200-239 mg/dL is defined to be borderline high, whereas a level ≥240 mg/dL is defined as high TC [3]. The awareness of the association between elevated LDL-C levels and CV risk has resulted in the creation of several guidelines [4,5] suggesting therapeutic agents for managing increased lipid levels, especially for reducing LDL-C and non-high-density lipoprotein cholesterol (HDL-C) cholesterol levels. Statins are the primary treatment of choice, but patients may experience intolerance, or be unable to reach their target LDL-C levels. This intolerance may be associated with myalgias or statin-induced myopathy. Sometimes, the patients’ inability to reach target LDL-C levels may be attributed to the subtherapeutic response. Plenty of factors, such as variations in the rate of drug absorption, metabolism, transport, excretion, or even in the levels of the non-kinetic target pathways could be attributed to subtherapeutic statin response [6]. Statins are the cornerstones of therapy [7] in combination with lifestyle changes for patients with clinical risk factors for CV diseases. High-intensity statin therapy is indicated for clinical ASCVD, but in cases of intolerance, moderate-intensity statins can be administered alternatively. If LDL-C levels remain ≥70 mg/dL despite maximally tolerated statin doses, then the addition of ezetimibe may be considered due to its promising results in the form of LDL-C reductions and better CV outcomes [7,8]. An LDL cholesterol target of 1.4 mmol/L has been identified [5] for patients at high to very high risk, but it may be unachievable for all patients due to statin intolerance in 10-20% of the patients [7,9].

Atrophy of LDL receptors is promoted by the proprotein convertase subtilisin-kexin type 9 (PCSK9) enzyme, resulting in reduced bloodstream clearance of LDL-C [10]. It has also been identified that increased or decreased levels of LDL-C could be associated with gain- or loss-of-function mutations for PCSK9 [11,12]. Mutations of PCSK9 have been recognized as genetic markers of familial hypercholesterolemia (FH), whereas lower levels of LDL-C, as well as reduced risk of coronary heart diseases, were found in those with loss-of-function mutations [13]. A new therapeutic target for LDL-C reduction was identified upon inhibition of PCSK9 binding to LDL receptors on hepatocytes, which led to an increased density of the LDL receptors and elimination of LDL particles from the bloodstream. This inhibition would potentially complement the mechanism of statins since they contribute to higher PCSK9 levels at transcription, thereby reducing statin resistance [14]. This led to guideline-based recommendations for the consideration of PCSK9 inhibitors among patients with high CV risks and treatment-resistant LDL-C levels despite receiving maximally tolerated statin doses combined with ezetimibe [8]. The addition of PCSK9 inhibitors to statin therapy has been reported to cause 43-64% reductions in LDL-C levels, but evaluations are still pending for their long-term safety [4]. PCSK9 inhibitors may also be considered if patients have LDL-C levels persistently ≥70 mg/dL, if the benefits are deemed to outweigh the risks, and if costs are not prohibitive [4].

In phase 2 clinical trials, two fully human monoclonal antibody (MAB)-based PCSK9 inhibitors, namely evolocumab (Repatha®) and alirocumab (Praluent®) were found to be well-tolerated across a diverse subject population and showed a dose-dependent decrease in LDL-C levels with no adverse incidents [15]. Phase 2 and phase 3 randomized controlled trials of evolocumab and alirocumab showed a ≤60% decrease in LDL-C levels when used in combination with maximally tolerated statins along with lifestyle changes in patients with FH and/or clinical ASCVD [16]. This resulted in an expedited Food and Drug Administration (FDA) approval for these agents [2]. Alirocumab was approved in 2015, and evolocumab was approved in 2016 [17]. Both agents are administered subcutaneously. Across all phase 3 studies, evolocumab was dosed at 140 mg every two weeks or 420 mg every month. On the other hand, alirocumab was dosed at 75 mg every two weeks and would be increased to a 150-mg dose every two weeks if LDL-C levels persisted at ≥70 mg/dL. Additionally, alternate regimens of alirocumab comprised of a 300-mg dose every four weeks and a 150-mg dose every two weeks without initial low-dose regimens [17]. A humanized antibody, bococizumab, was also clinically tested for its safety and efficacy for PCSK9 inhibition. During the later stages of the development, antibodies neutralizing bococizumab were found to affect the long-term implications of the drug on LDL-C levels, and hence further developmental plans were put on hold [17-19].

In this review, we evaluated the available literature and data from randomized studies to better understand the role played by MABs, mainly evolocumab and alirocumab, in the treatment and management of hypercholesterolemia.

## Review

Methods

Between December 2021 and January 2022, a literature search was conducted using PubMed/MEDLINE, ProQuest, Google Scholar, Elsevier, and Web of Science as the primary databases for this topic. Studies published in languages other than English were excluded from search results, along with studies not pertinent to the hypercholesterolemia and hyperlipidemia disease states. For relevance and convenient access, only full-text sources published between 2010 and 2022 were included. The JAMA Neurology website [20] was utilized to rank the quality of evidence. A higher rank was given to randomized controlled trials with good power, as well as to systematic reviews with meta-analysis, whereas opinions and narrative reviews were ranked lowest on the scale. The literature search elicited 34 relevant studies for analysis for this review, comprised of, but not limited to, randomized controlled clinical trials, meta-analyses, and cohort studies. The data from the 34 studies obtained via the literature search represented an aggregate of 178,627 patients.

Mechanism of action of PCSK9

PCSK9 is a ligand for LDL receptors and the identification of its function in metabolizing lipids has led to efforts for the development of novel therapies that could either reduce its levels in the body or inhibit it through protein synthesis or by binding with LDL receptors [21]. Free circulating PCSK9 results in a reduction of hepatic surface LDL receptors, causing an increase in LDL-C levels due to low clearance on account of lysosomal degradation of the receptors [13]. Unlike small therapeutic molecules, biologic therapies exhibit higher levels of target specificity and possess a lower potential for drug-to-drug interactions due to their elimination through the reticuloendothelial system. About 80% of human antibodies belong to the immunoglobulin G (IgG) subtype, and all of them are naturally produced by the B cells in the human body [13].

Monoclonal antibody-based PCSK9 inhibitors as monotherapy

In 614 patients with hypercholesterolemia (LDL-C levels of 100-190 mg/dL), evolocumab was evaluated as a monotherapy [17,22]. The inclusion of participants was based on LDL-C level and the absence of any prior history of pharmacologic treatment for dyslipidemia, whereas patients with a history of coronary artery disease, diabetes, or any other diseases were excluded from participation. Treatment arms included administration of oral ezetimibe or placebo and subcutaneous evolocumab or placebo in a randomly assigned manner. After 12 weeks, a 55% reduction in LDL-C from a mean baseline of 143 mg/dL was seen with the administration of monthly evolocumab, whereas weekly administration of the agent resulted in a 57% reduction in LDL-C from the mean baseline.

In 103 patients with moderately increased CV risk and no prior history of dyslipidemia treatment, alirocumab was used as monotherapy for LDL-C levels of 100-190 mg/dL. A 75-mg dose was administered subcutaneously every two weeks and was increased to 150 mg/dL at the beginning of the 12th week if LDL levels persisted at ≥70 mg/dL in the eighth week. A 47% reduction in LDL-C level was seen in the intention-to-treat group, and a 53% reduction was observed in the treatment group after 24 weeks [17]. Additionally, a 52% reduction in LDL-C was seen at week 24 in another study that evaluated the efficacy of a 300-mg dose of alirocumab administered every four weeks [22]. This data became the basis for the FDA's approval of both therapies for the reduction of high LDL-C levels, as highlighted in the 2018 AHA guidelines for the treatment of hypercholesterolemia [4].

In a multicenter phase 2 study - monoclonal antibody against PCSK9 to reduce elevated LDL-C in subjects currently not receiving drug therapy for easing lipid level (MENDEL) - conducted [9] across 52 centers in Europe, the United States, Canada, and Australia, 406 patients were randomly assigned to one of of the following nine interventions: biweekly, subcutaneous injections of evolocumab 70 mg, 105 mg, 140 mg, or placebo; subcutaneous injections of evolocumab 280 mg, 350 mg, or 420 mg administered every four weeks, or placebo; or a 10-mg daily oral dose of ezetimibe. A considerable, dose-dependent decrease in LDL-C levels from baseline was observed at week 12 across all the evolocumab patient groups versus the placebo groups (p<0.00001). Additionally, at week 12, all evolocumab groups showed considerably higher mean changes from baseline LDL-C levels (-14.7%) compared to the ezetimibe groups (95% CI: -18.6 to -10.8; p<0.0001). Notable reductions in free PCSK9 levels were also observed from all evolocumab treatment doses, along with a minor decrease in triglyceride levels, and a significant increase in HDL-C levels [9]. This was a monotherapy-based trial and patients were not required to have a history of statin intolerance. All subcutaneous treatment options and doses were administered in a double-blind fashion, apart from ezetimibe, which could not be masked due to its oral administration. Of note, 63% of patients were assigned to either the evolocumab treatments or the subcutaneous placebo, or ezetimibe, and most of the remaining patients were provided all the planned interventions [9].

A phase 3, randomized, MENDEL-2 trial was carried out in 614 patients with LDL-C levels of 100-190 mg/dL. The patients were given a weekly oral and subcutaneous placebo, or monthly oral and subcutaneous placebo, or weekly oral ezetimibe combined with a subcutaneous placebo, or oral placebo combined with biweekly 140-mg subcutaneous evolocumab, or an oral placebo combined with monthly 420-mg subcutaneous evolocumab in a 1:1:1:1:2:2 ratio. At the end of the trial, evolocumab was found to have led to significantly decreased LDL-C levels from baseline by 55-57% versus placebo and by 38-40% versus ezetimibe (p<0.001 across all groups). Lower lipoprotein levels were also seen with the use of evolocumab [23].

Monoclonal antibody-based PCSK9 inhibitors as part of a combination therapy

The ODYSSEY COMBO I and II trials [13] were pioneer studies where alirocumab was studied as an add-on treatment in hypercholesterolemia patients on maximally tolerated statin doses in combination with other lipid-lowering agents. The primary endpoint in both the studies was the distinction between both intervention groups in terms of percentage change in LDL-C from baseline to week 24 utilizing all LDL-C levels regardless of the patient’s compliance to the therapy in an intention-to-treat approach [13]. Median baseline free and total PCSK9 levels were found to be higher among patients on prior statin therapy, and a higher baseline LDL-C level of 140.4 mg/dL was found among patients who were given a higher dose of biweekly alirocumab (150 mg) versus that of 101.1 mg/dL in patients who continued to receive the 75 mg biweekly dose of alirocumab. By week four of the study, an increase in concentration was seen for alirocumab, whereas LDC-L levels had decreased in association with lower concentrations of free PCSK9 (108.2-154.6 ng/mL in the COMBO II study) and were maintained throughout the course of the study. These changes were also observed to be associated with the increment in the number of inactive PCSK9-alirocumab complexes [24].

Both COMBO I and II were phase 3, randomized, double-blind, parallel-group trials - except that the COMBO I trial was placebo-controlled and conducted in 76 sites within the United States, whereas the COMBO II trial was active-controlled and conducted in 126 sites across Europe, Israel, North America, South Africa, and South Korea. COMBO II also comprised a double-dummy design where patients were given placebo ezetimibe or placebo alirocumab, and patients were only allowed to receive their prior statin regimens in combination with their randomized therapy. All patients in the COMBO I and II trials had a history of hypercholesterolemia and coronary heart disease or equivalent risk factors. These included peripheral arterial disease, ischemic stroke, chronic kidney disease, diabetes mellitus, hypertension, and an ankle-brachial index ≤0.90. LDL-C levels were uncontrolled prior to trials despite patients having been on maximally tolerated statin doses [13]. Additionally, the COMBO I subject population was allowed to receive other lipid-lowering medications in combination with statins if both the medications were administered at a stable dose for a minimum of four weeks before the screening period. On the other hand, the COMBO II trial did not allow any additional lipid-lowering medications other than stable doses of statins for a minimum of four weeks before the screening period. In the COMBO I study, patients were randomized in a 2:1 fashion with alirocumab and placebo and a double-blind treatment period of 52 weeks was started afterward. In the COMBO II study, patients were randomized in a 2:1 fashion to alirocumab and ezetimibe in a double-blind, double-dummy phase where they either received a combination of subcutaneous, biweekly 75-mg alirocumab and a daily 10-mg oral placebo of ezetimibe, or a combination of subcutaneous, biweekly placebo for alirocumab and a 10-mg daily oral dose of ezetimibe [13].

The ODYSSEY trial [11] also entailed a major CV outcomes study that investigated the long-term effects of alirocumab on LDL-C reduction, and their association with CV events in a patient population of 18,924 with a history of recent acute coronary syndrome episodes within the previous year. A composite primary endpoint event (death from coronary heart disease, nonfatal myocardial infarction, any type of ischemic stroke, or unstable angina needing hospital admission) was reported in 11.1% of patients in the placebo group and in 9.5% of patients in the treatment arm [hazard ratio (HR): 0.85; 95% confidence interval (CI): 0.78-0.93; p<0.001]. Deaths were reported among 3.5% of patients in the treatment group, whereas 4.1% of patients died in the placebo group. Patients with a baseline LDL-C level ≥100 mg/dL showed more benefit from alirocumab in relation to the primary composite endpoint, and a reduced risk of recurring CV events was witnessed among patients receiving alirocumab versus those who were administered the placebo [11]. This was a multicenter, randomized, double-blind, placebo-controlled clinical trial where 50% of patients were randomly assigned to a biweekly, subcutaneous 75-mg dose of alirocumab, and the remainder was assigned to a matching placebo administered in a similar fashion as the active treatment. 

A long-term phase 3 trial (ODYSSEY LONG TERM) was also conducted to assess the long-term safety and efficacy of alirocumab in patients with high CV risk and poorly controlled LDL-C levels despite a maximally tolerated statin dose regimen [25]. For 78 weeks, 2,341 patients were randomized in a 2:1 ratio, to be given either 150-mg subcutaneous alirocumab or placebo every two weeks. In terms of the primary efficacy endpoint of percentage change in LDL-C levels from baseline to week 24, the observed difference was -62% points, which was statistically significant (p<0.001). Regarding safety events, higher instances of injection-site reactions, myalgia, neurocognitive events, and ophthalmologic events were observed across the alirocumab groups versus placebo groups. A post-hoc analysis observed lower rates of major CV events with alirocumab (1.7%) versus placebo (3.3%) (HR: 0.52; 95% CI: 0.31-0.90; p=0.02). Hence, treatment of hypercholesterolemia with alirocumab in addition to maximally tolerated statin dose may considerably lower LDL-C concentrations and could result in lower instances of CV events [26].

The Further Cardiovascular Outcomes Research with PCSK9 Inhibition in Subjects with Elevated Risk (FOURIER) trial [17] was a pioneer phase 3 study conducted among patients with a history of ASCVD (myocardial infarction, non-hemorrhagic stroke, or symptomatic peripheral artery diseases presenting with additional risk factors) on a regimen of a 20-mg minimum daily dose of atorvastatin and LDL-C levels >70 mg/dL. An absolute decrease of 62 mg/dL in LDL-C levels was observed in 59% of the evolocumab treatment population versus the placebo group at 48 weeks. A significant reduction was observed in the primary composite endpoint for components of myocardial infarction, stroke, and coronary revascularization, but no significant decreases were observed for the risks related to unstable angina, and CV or all-cause mortality [17]; 27,654 patients with a median baseline LDL-C level of 92 mg/dL were given evolocumab or subcutaneous placebo in a randomized, 1:1 manner, where they received evolocumab in a biweekly subcutaneous dose of 140 mg or a 420 mg monthly dose, or a placebo. During the study period, a target LDL-C level of <70 mg/dL was recommended by most dyslipidemia guidelines, but a secondary analysis assessing the effect of evolocumab on patients with LDL-C more or less than this range reported no major differences in CV benefits between both populations. Evolocumab also showed benefits irrespective of prior statin regimens [17].

To assess the neurological safety of evolocumab, a sub-study EBBINGHAUS was conducted under the FOURIER trial, where low LDL-C levels were found to have no associations with cognitive changes [17]. Another 52-week-long, phase 3 trial of evolocumab was carried out across nine countries in a study group of 901 patients who had LDL-C levels ≥75 mg/dL. In a 2:1 ratio, the participants were randomized to be given either a 420-mg subcutaneous dose of evolocumab or a placebo every four weeks and were assessed at week 52 for the primary endpoint of percentage change from baseline LDL-C levels. A statistically significant (p<0.001) mean reduction of 57.0 ±2.1% was seen in the evolocumab group. Significantly lower levels of apolipoprotein B, non-HDL cholesterol, lipoprotein (a), and triglycerides were also observed with the use of evolocumab. In the evolocumab group, nasopharyngitis was the most observed adverse event along with upper respiratory tract infection, influenza, and back pain [27].

A study named Open-Label Study of Long Term Evaluation Against LDL-C (OSLER-1) [28] was carried out to assess the long-term impact of evolocumab in an open-label hypercholesterolemia treatment for a duration of five years - the longest period for the use of PCSK9 inhibitor antibodies. Under this trial, patients who participated in any of phase 2 or phase 3 evolocumab studies were randomized in a 2:1 fashion to receive the guideline-based standard treatment or a 420-mg monthly dose of subcutaneous evolocumab for the first year and would later be initiated into a four-year period of receiving evolocumab in addition to guideline-based standard treatment. A total of 1,255 patients enrolled in the first year of the study, out of which 1,151 patients entered the four-year duration of the all-evolocumab treatment. The primary endpoint of the study was to assess the safety and tolerability of long-term use of evolocumab. The open-label study also evaluated additional safety aspects of the treatment, such as annual instances of adverse events leading to discontinuation of treatment, and the presence or absence of anti-drug antibodies in the subject population. For the secondary endpoint, the percentage change in LDL-C levels was taken into account, along with HDL-C, total cholesterol, triglycerides, apolipoproteins A1 and B, and lipoprotein (a). It was found that LDL-C levels decreased from 120 mg/dL to 48 mg/dL (p<0.001) through evolocumab treatment without any serious safety events [28]. No instances of anti-drug antibody formation were found for evolocumab throughout the duration of the long-term study. The results of OSLER-1 seemed to corroborate those from the FOURIER study in that no association was found between cognitive changes and long-term treatment with evolocumab, suggesting no increased risk of development of those events even with prolonged administration of evolocumab. Additionally, a decrease was seen in injection-site reactions in the years two to five of treatment compared to that in the first year of the study (0.2% versus 4.1%), and for the frequency of hypersensitivity reaction events (10.2% in the first year versus 7.3% in the fifth year). The findings from the study indicated the long-term safety and efficacy of evolocumab over a period of five years [29].

The safety profile of monoclonal antibody-based PCSK9 inhibitors

While abnormal liver function and muscle enzyme levels may be attributed to statin therapy, considerable assessments have been made to analyze the safety of the two approved PCSK9 inhibitors: evolocumab and alirocumab. Due to the antibodies being fully human in nature, a low potential for immunogenicity has been identified, resulting in lower instances of immune system reactions to alirocumab and evolocumab [30]. Besides injection-site reactions, very few instances of adverse effects were noticed in the trials for both the agents. LDL-C metabolism is also associated with vitamin E transport and an absolute reduction in vitamin E levels was seen in a randomized trial of 901 patients, where it decreased by 16% from baseline to week 52 in patients treated with evolocumab but showed a 19% increase under normal cholesterol levels [21].

Future directions

In the treatment setting, PCSK9 inhibitor antibodies may find a useful place as part of the existing guideline recommendations for the treatment of hypercholesterolemia in patients with or without clinical ASCVD needing additional LDL-C reductions despite statin regimens. The longer biweekly dosing intervals seen for alirocumab as well as monthly and biweekly dosing for evolocumab may have a key function in regulating patient compliance to these treatments. Self-administration may also help in maintaining patient adherence to therapy but may require the patient to self-inject or the presence of a caregiver for administration. When prescribed to patients in whom the reduction of risk is increased under reduced LDL-C levels, higher costs associated with these PCSK9 inhibitor antibodies may be justified [1].

Table 1 below summarizes a few major trials and their findings on the safety and efficacy of evolocumab and alirocumab.

**Table 1:** Summary of important study findings on the safety and efficacy of evolocumab and alirocumab PCSK9: proprotein convertase subtilisin/kexin type 9; LDL-C: low-density lipoprotein cholesterol; HDL-C: high-density lipoprotein cholesterol; ASCVD: atherosclerotic cardiovascular diseases

Study	Key findings	Importance
Efficacy, safety, and tolerability of a monoclonal antibody to proprotein convertase subtilisin/kexin type 9 as monotherapy in patients with hypercholesterolemia (MENDEL): a randomized, double-blind, placebo-controlled, phase 2 study [9]	Evolocumab caused a significant dose-dependent decrease in LDL-C levels from baseline at week 12, along with notable reductions in free PCSK9 levels, and a minor decrease in triglyceride levels. A significant increase in HDL-C levels was observed in the evolocumab patients	The data from this study became the basis of the phase 3 MENDEL-2 study evaluating the long-term safety and efficacy of evolocumab in a more diverse population with a history of statin intolerance
Monoclonal antibody against PCSK9 to reduce elevated LDL-C in subjects currently not receiving drug therapy for easing lipid levels-2 (MENDEL-II) [23]	55% reduction in LDL-C levels from baseline after 12 weeks of evolocumab as a monthly monotherapy. 57% reduction in LDL-C levels from baseline when evolocumab was administered weekly	Largest monotherapy trial where evolocumab was found to be well-tolerated and caused significant LDL-C reductions versus ezetimibe or placebo
A 52-week placebo-controlled trial of evolocumab in hyperlipidemia: the durable effect of PCSK9 antibody compared with placebo study (DESCARTES) [27]	In the evolocumab group, at 52 weeks, LDL-C levels were reduced by 57.0 ±2.1% (p<0.001). Evolocumab significantly reduced levels of apolipoprotein B, non-HDL cholesterol, lipoprotein (a), and triglycerides	Evolocumab caused significant LDL-C reduction in patients on statin therapy who were at risk for coronary diseases
Efficacy and safety of evolocumab in reducing lipids and cardiovascular events: the open-label study of long-term evaluation against LDL cholesterol (OSLER) I and II studies [28]	Evolocumab reduced the LDL-C levels from a baseline of 120 mg/dL to 48 mg/dL. Years 2-5 of treatment with evolocumab showed lower instances of injection-site reactions versus year 1	The longest trial evaluating the use of PCSK9 inhibitor antibodies. Findings indicated the long-term safety and efficacy of evolocumab [30]
Efficacy and safety of alirocumab in reducing lipids and cardiovascular events: the ODYSSEY LONG TERM trial [25]	Alirocumab caused a 62% reduction in LDL-C levels at week 24 compared to placebo. Post hoc analysis observed lower rates of major cardiovascular events with alirocumab	Consistent reductions in LDL-C levels were seen with alirocumab over 78 weeks, along with a reduction in cardiovascular events in patients on maximally tolerated statin doses
A phase III randomized trial evaluating alirocumab 300 mg every 4 weeks as monotherapy or add-on to statin: ODYSSEY CHOICE I [22]	52% reduction in LDL-C levels from baseline after 24 weeks of a 300m g dose of alirocumab administered every 4 weeks	Alirocumab 300 mg Q4 weeks may be a useful therapeutic choice with or without statins in patients who need additional options for LDL-C lowering
Evolocumab and clinical outcomes in patients with cardiovascular disease: the FOURIER trial [31]	A 62% reduction in LDL-C levels was observed in 59% of the evolocumab patients. In patients on statin therapy, PCSK9 inhibition from evolocumab reduced LDL-C levels to a median of 30 mg/dL. A significant reduction was observed in the primary composite endpoint for components of myocardial infarction, stroke, and coronary revascularization	Patients with ASCVD benefit from LDL-C reductions below current targets
Alirocumab and cardiovascular outcomes after acute coronary syndrome: the ODYSSEY OUTCOMES trial [32]	Patients with baseline LDL-C levels ≥100 mg/dL showed a greater benefit with alirocumab in terms of the composite primary endpoint of death from coronary heart disease, nonfatal myocardial infarction, fatal or nonfatal ischemic stroke, or unstable angina requiring hospitalization	Alirocumab was associated with a lower risk of recurrent ischemic cardiovascular events versus placebo when administered to patients with a history of acute coronary syndrome and on a high-intensity statin regimen
Efficacy and safety of the proprotein convertase subtilisin/kexin type 9 inhibitor alirocumab among high cardiovascular risk patients on maximally tolerated statin therapy: The ODYSSEY COMBO I study [33]	At week 24, 75% of the alirocumab group patients achieved LDL levels <70 mg/dL	At 24 weeks, alirocumab therapy led to a higher proportion of patients attaining their target LDL-C levels with inadequately controlled hypercholesterolemia despite maximally tolerated statin therapy with or without additional lipid-lowering treatment choices
Efficacy and safety of alirocumab in high cardiovascular risk patients with inadequately controlled hypercholesterolemia on maximally tolerated doses of statins: the ODYSSEY COMBO II randomized controlled trial [34]	At week 24, 77% of patients on alirocumab achieved LDL-C levels <1.8 mmol/L	Alirocumab was found to be well-tolerated and caused notably higher reductions in LDL-C levels versus ezetimibe in patients on statin therapy

## Conclusions

Hypercholesterolemia is a prevalent condition that could lead to high rates of atherosclerotic cardiovascular diseases (ASCVD) globally. Statin drugs are the therapy of choice in reducing LDL-C levels and improving ASCVD risk in this patient population. By binding onto LDL receptor sites on hepatocytes, the proprotein convertase subtilisin/kexin type 9 (PCSK9) enzyme causes an increase in LDL-C levels, and a reduction in LDL-C results from the inhibition of the PCSK9 enzyme. Although PCSK9 inhibitor antibodies represent a recent avenue of combination treatment with statins and ezetimibe for hypercholesterolemia in patients with treatment-resistant LDL-C levels and/or a history of statin intolerance, their clinical benefits in these patients appear to be very promising. Additionally, the favorable safety profiles and convenient dosing intervals for alirocumab and evolocumab may be beneficial for patients’ adherence to therapy. These factors may play a key role in the development of PCSK9 inhibitor antibodies as the third cornerstone of therapy in this disease state. However, it may be of interest to see a randomized, multicenter, double-blind study that could assess the long-term safety and efficacy of alirocumab and evolocumab, which would aid in making clinical treatment decisions.
